# Melatonin mitigates bisphenol A- and MBP-induced damage in Leydig cells by alleviating oxidative stress and endoplasmic reticulum stress via inhibition of PERK signaling pathway

**DOI:** 10.1016/j.cstres.2026.100222

**Published:** 2026-10-15

**Authors:** Qi Qi, Jijing Xu, Shuang Li, Xiaoyan Pan

**Affiliations:** Center for Reproductive Medicine, Jilin Medical University, Jilin, 132013, PR China

**Keywords:** Melatonin, BPA, BPA metabolites, Leydig cells, SIRT1, PERK signaling pathway, Endoplasmic reticulum stress

## Abstract

This study aims to elucidate the protective effects of melatonin against Bisphenol A (BPA) and its metabolite 4-methyl-2,4-bis(4-hydroxyphenyl)pent-1-ene (MBP)-induced apoptosis in Leydig cells, focusing on its ability to alleviate oxidative stress and endoplasmic reticulum stress. Results showed that melatonin significantly alleviated testicular damage caused by BPA, increased testicular index in mice, improved the function of seminiferous tubules, and enhanced the survival rate and density of epididymal sperm. Furthermore, melatonin treatment significantly reduced the number and proportion of TUNEL-positive Leydig cells, decreased cleaved caspase-3 expression and the cleaved caspase-3/total caspase-3 ratio, increased testosterone secretion from Leydig cells, and elevated the expression of steroidogenic enzymes cytochrome P450 17α-hydroxylase/20-lyase and 17β-hydroxysteroid dehydrogenase 3. Melatonin also significantly lowered the elevated ROS levels in Leydig cells induced by MBP and enhanced the expression of antioxidant enzymes superoxide dismutase 1/2 and catalase. Additionally, melatonin effectively countered endoplasmic reticulum stress in Leydig cells induced by BPA and MBP, significantly decreasing levels of GRP94 and GRP78, while inhibiting activation of the PERK/EIF2α/ATF4/CHOP signaling pathway. Notably, melatonin significantly alleviated endoplasmic reticulum stress and apoptosis in TM3 cells, and this effect was associated with enhanced SIRT1 nuclear localization and deacetylase activity, as demonstrated by reduced acetylated-p53 levels. Collectively, melatonin effectively protects Leydig cells from BPA and MBP-induced damage by alleviating oxidative stress and endoplasmic reticulum stress through inhibition of the PERK signaling pathway, an effect that may involve SIRT1 activation. It may serve as a therapeutic agent to safeguard male reproductive health against environmental pollutants.

## Background

In recent decades, environmental endocrine disruptors and their metabolites have emerged as significant harmful substances affecting human health.^1^, ^2^ Bisphenol A (BPA) is a major and ubiquitous endocrine disruptor found in epoxy resins, polycarbonate plastics, thermal paper dyes, food packaging, aerosol particles, and skincare products. It can induce reproductive toxicity, particularly affecting male reproductive health.^3^ Mechanically, it induces oxidative stress in testicular cells, particularly influencing Leydig cells, which results in their apoptosis and a reduction in testosterone synthesis.^4^, ^5^ Oxidative stress causes the accumulation of reactive oxygen species (ROS) within cells, which can disrupt protein folding in the endoplasmic reticulum, triggering the unfolded protein response (UPR) and inducing endoplasmic reticulum stress (ERS),^6^ ultimately leading to cell apoptosis. 4-methyl-2,4-bis(4-hydroxyphenyl)pent-1-ene (MBP) is a significant active metabolite of BPA generated through liver metabolism. It has been reported that the toxic effect of MBP on medaka (Oryzias latipes) is approximately 250 times greater than that of BPA.^7^ In neuronal cells, MBP demonstrates greater cytotoxicity than BPA, capable of inducing mitochondria-dependent and ERS-triggered apoptosis.^8^ Therefore, oxidative stress and ERS may be the primary pathways through which BPA and MBP exert adverse effects on the body and cells.

Antioxidants play a protective role in mitigating BPA-induced cytotoxicity.^9^, ^10^ Melatonin (MT), a naturally occurring hormone recognized for its antioxidant properties, has been shown to inhibit apoptosis in various cell types by alleviating the adverse effects of oxidative stress and ERS.^11^, ^12^, ^13^ A primary mechanism through which MT exerts its protective effects is the regulation of the Sirtuin 1 (SIRT1) pathway.^11^, ^14^, ^15^ SIRT1, an NAD^+^-dependent deacetylase, plays a critical role in maintaining cellular homeostasis by regulating multiple signaling pathways associated with stress responses, metabolism, and apoptosis.^11^ It can alleviate ERS by inhibiting the activation of the PERK (protein kinase RNA-like endoplasmic reticulum kinase) / EIF2α (eukaryotic translation initiation factor 2 alpha) / CHOP (C/EBP homologous protein) signaling pathway, thereby reducing apoptosis and cell death.^14^ MT increases the intracellular levels of the antioxidant enzyme catalase (CAT) through the SIRT1 pathway, leading to reduced ROS levels in porcine Leydig cells and alleviating both apoptosis and decreased testosterone secretion in these cells.^16^ However, it remains unclear whether MT can ameliorate MBP-induced apoptosis in Leydig cells via the SIRT1 and PERK signaling pathway and what specific regulatory mechanisms are involved.

This study aims to investigate the effects of MT on BPA- and MBP-induced oxidative stress and ERS in Leydig cells through both *in vivo* and *in vitro* experiments. Additionally, it seeks to elucidate the mechanistic role of SIRT1 and PERK signaling in mediating the protective effects of MT against BPA- and MBP-induced damage. The findings of this research will provide a theoretical foundation and experimental evidence for mitigating the adverse effects of these common pollutants on male fertility and overall reproductive health.

## Methods

### Experimental animals

This study used sexually mature male Kunming mice (*N* = 40; body weight 35 ± 2 g) (Yisi Experimental Animal Technology Co., Ltd., Changchun, China). The mice were housed in a temperature-controlled environment (22°C ± 2°C) with a 12-h light/12-h dark cycle. They were provided with ad libitum access to water and food. All handling procedures for the mice were approved by the Ethics Committee of Jilin Medical University (approval no. 2022-KJT016). All animal studies complied with the ARRIVE guidelines and the AVMA euthanasia guidelines 2020.

## Drug preparation

BPA (239658, Sigma-Aldrich) was dissolved in olive oil to achieve a concentration of 20 mg/mL. For animal experiments, MT (M5250, Sigma-Aldrich) was dissolved in a mixture of ethanol and normal saline at a ratio of 1:9 to prepare an 8 mg/mL MT solution. For cell experiments, MBP (S706213, Sigma-Aldrich) or MT (M5250, Sigma-Aldrich) was dissolved in DMSO to create a concentrated stock solution at 1 mM. This concentrated stock solution was then diluted with DMEM/F12 medium (11320033, Gibco) to yield working solutions of various concentrations as required. Similarly, thapsigargin (TG) (HY-13433, Med Chem Express, NJ, USA) and EX-527 (HY-15452, Med Chem Express) were also dissolved in DMSO to prepare a stock solution at 1 mM, and subsequent dilution with DMEM/F12 medium was performed to yield working solutions of 500 nM TG or 10 μM EX-527.

### Animal grouping and treatment

Mice were randomly divided into four groups (*N* = 10 per group): the control group, BPA group, MT group, and BPA + MT group. Mice in the BPA and BPA + MT groups received daily gavage of 50 mg/kg BPA, which corresponds to the lowest observed adverse effect level in mice as defined by the U.S. Environmental Protection Agency BPA Action Plan (CASRN 80–05–7). This dose is well-established for inducing experimental testicular injury and enables reproducible assessment of pharmacological interventions.^17^, ^18^ This dose stably induces significant apoptosis of testicular Leydig cells within the 30-day intervention period while avoiding interference from severe necrosis, making it suitable for quantitative apoptosis assessment. Mice in the control and MT groups received the same volume of olive oil daily. Meanwhile, mice in the MT and BPA + MT groups received intraperitoneal injections of 20 mg/kg MT every other day. This dose was selected based on our previous dose-response study demonstrating superior testicular protection of 20 mg/kg MT compared to 10 mg/kg MT in BPA-exposed mice.^5^ Mice in the control and BPA groups were injected with an equal volume of normal saline every other day. After 30 days of drug intervention, the testes and epididymis of the mice were collected, and the testicular index (the ratio of testis weight to body weight) was calculated. The right testis was preserved in Bouin’s solution (BL-GO16, SenBeiJia Biological Technology Co., Ltd., Nanjing, China) for fixation for 24 h, while the left testis was stored at −80°C in a freezer. The control mice and the experimental mice were treated simultaneously, and samples were collected synchronously.

### HE staining

After fixation, the testes of the mice were dehydrated in a gradient ethanol solution, treated with xylene for 15 min, and subsequently embedded in paraffin. The testicular tissue was sectioned into 5 μm-thick slices, subjected to xylene and gradient ethanol treatment, and stained with hematoxylin (S0152, Bioss Biotechnology, Beijing, China) for 4 min, followed by eosin (C145380, Sino Biological, Beijing, China) for 1 min. The sections were then mounted with neutral resin, and the structure of the testicular tissue was observed and photographed using an Olympus BX53 microscope (Tokyo, Japan).

### Sperm motility and density analysis

The cauda epididymis was placed into 1 mL of pre-warmed PBS buffer at 37°C and punctured using a 26G needle, followed by incubation at 37°C for 15 min. A 150 μL aliquot of the sperm suspension was obtained, mixed with 10 μL of a 1% eosin solution, and a sperm smear was prepared. The staining of the sperm heads was observed under an Olympus IX83 microscope. Dead sperm were stained red, while live sperm remained unstained. The number of live and dead sperm was counted across five mice per group to calculate the sperm survival rate. For sperm density analysis, the sperm suspension was diluted with a 3% NaCl solution (1:10), and 10 μL of the diluted sperm suspension was counted on a hemocytometer to calculate sperm density (sperm/mL).

### Cell line and culture

The TM3 mouse Leydig cell line (CL-0234, Wuhan Punosai Life Technology, Wuhan, China) was cultured in DMEM/F12 medium (11320033, Gibco) supplemented with 10% fetal bovine serum (BL201A, Biosharp, Hefei, China) and 1% penicillin-streptomycin (P1400, Solarbio, Beijing, China). The cells were passaged every 2 days at 37°C under 5% CO_2_ conditions, maintaining a confluence of 80%-90%.

### Cell treatment

The TM3 cells were divided into the control group, MBP group, MBP + MT group, TG group, TG + MT group, EX527 group, and MBP + EX527 + MT group. The control group cells were cultured in medium for 32 h. Cells in the MBP group were first cultured in medium for 8 h and then treated with MBP-supplemented medium for 24 h. Cells in the TG group were similarly cultured for 8 h before being treated with TG-supplemented medium for 24 h.^19^ The EX527 group cells were treated with EX527 for 2 h,^20^ followed by culturing in medium for 30 h. In the MBP + MT group, cells were cultured in medium for 2 h before receiving MT treatment for 6 h, followed by MBP treatment for an additional 24 h.^18^ In the TG + MT group, cells were similarly cultured for 2 h, then treated with MT for 6 h, after which TG was administered for 24 h. Cells in the MBP + EX527 + MT group were treated with EX527 for 2 h, then with MT for 6 h, and finally with MBP for 24 h. During cell culture, the concentrations of MBP, MT, TG, and EX527 added were 13 μM, 5 μM, 500 nM, and 10 μM, respectively. The MBP concentration (13 μM) was selected based on its IC_50_ value (12.96 μM) in TM3 cells, as previously determined by CCK-8 assay.^18^ This concentration was chosen to induce substantial apoptosis while preserving a sufficient number of viable cells for evaluating the protective effects of MT. MT (5 μM) was used based on dose-response experiments in TM3 cells demonstrating optimal cytoprotective effects.^18^ TG (500 nM) was employed to induce ERS following established protocols in Leydig cells.^19^ EX527 (10 μM) was used to inhibit SIRT1 activity based on previous studies in Leydig cells,^20^ and its efficacy was confirmed by monitoring acetylated-p53 levels.

### CCK-8 assay

The CCK-8 kit (C0038, Beyotime, Shanghai, China) was used to evaluate cell viability and drug-induced growth inhibition. Briefly, TM3 cells were seeded in a 96-well plate at a density of 5000 cells per well, with seven replicates for each group. The cells were incubated in DMEM/F12 medium for 12 h to facilitate adequate adhesion. Then, the cells were grouped and treated as described above. After that, 20 μL of CCK-8 solution was added to each well. After an additional 1-h incubation, the optical density (OD) at 450 nm of each well was measured using a microplate reader (CMax Plus, Molecular Devices, Shanghai, China). Cell viability was calculated with the following formula: Cell viability (%) = (OD value of experimental group − OD value of blank control group)/(OD value of control group − OD value of blank control group) × 100%.

### TUNEL assay

The TUNEL apoptosis detection kit (MK1019, Boster Biological Engineering, Wuhan, China) was used to assess DNA fragmentation in testicular tissue and TM3 cells. In detail, TM3 cells were fixed at room temperature with 4% paraformaldehyde for 30 min. Paraffin-embedded testicular tissues were digested with Proteinase K at 37°C for 15 min. Subsequently, the cell and tissue samples were treated with a buffer containing terminal deoxynucleotidyl transferase and digoxigenin-labeled dUTP for 2 h. Subsequently, 5% BSA was added for blocking at room temperature for 30 min. After blocking, the cell and tissue samples were sequentially incubated with biotinylated digoxigenin antibody and FITC-conjugated streptavidin. Both incubations were performed at 37°C for 30 min. Finally, Hoechst 333258 (C1018, Beyotime) was added for a 5-min incubation at room temperature to stain the nuclei. The positive control used was intestinal tissue. Staining was observed under Olympus IX73 and Olympus BX53 microscopes, where TUNEL-positive cells displayed bright yellow-green fluorescence. The number and ratio of TUNEL-positive cells were analyzed using ImageJ software.

### Immunofluorescence staining

Deparaffinized testicular tissue sections were subjected to antigen retrieval in EDTA antigen retrieval solution (RA0023, Bioss) for 10 min. After blocking with 5% BSA for 30 min, rabbit-derived antibodies against glucose-regulated protein 78 (GRP78) (A23453, ABclonal, 1:100), glucose regulated protein 94 (GRP94) (A26311, ABclonal, 1:100), cleaved caspase-3 (A27145, ABclonal, 1:50), CHOP (A72872, NatureBiosciences, 1:100), SIRT1 (A95664, NatureBiosciences, 1:100), and acetylated-P53 (AMM00772, EnkiLife, 1:100) were added to the tissue sections and incubated overnight at 4°C. Following washing, FITC-conjugated goat anti-rabbit secondary antibodies (A0562, Beyotime) were applied to the sections and incubated in the dark at 37°C for 1 h. DAPI staining was performed for 5 min to visualize the nuclei. The staining was observed and photographed using an Olympus BX53 microscope. The quantity of cleaved caspase-3-positive cells and the average fluorescence intensity of GRP78 and GRP94 in the testicular interstitial tissue were analyzed using ImageJ software.

### Flow cytometry detection of apoptosis

The Annexin V-FITC apoptosis detection kit (C1062L, Beyotime) was used to assess apoptosis in TM3 cells. Briefly, TM3 cells (8 × 10^4^) were collected and mixed with binding buffer. Subsequently, 5 µL of Annexin V-FITC and 10 µL of propidium iodide staining solution were added, followed by incubation in the dark at room temperature for 15 min. Apoptosis was detected using a flow cytometer (NovoCyte, ACEA Biosciences).

### ROS detection

ROS levels in TM3 cells were measured using a ROS detection kit (S0033S, Beyotime). The cultured cells were washed with PBS and then incubated with the fluorescent probe DCFH-DA at a concentration of 10 µM at 37°C for 20 min. Cells treated with Rosup served as a positive control. The cells were washed three times with serum-free DMEM/F12 medium and observed under an inverted fluorescence microscope (IX83, OLYMPUS), where cells expressing ROS exhibited green fluorescence. The average fluorescence intensity of ROS was analyzed using ImageJ software.

### ELISA

The ELISA kits for testosterone (#YJ001948), superoxide dismutase 1 (SOD1) (#YJ34719), superoxide dismutase 2 (SOD2) (#YJ35239), CAT (#YJ34657), cytochrome P450 17A1 (CYP17A1) (#YJ37826), and 17β-hydroxysteroid dehydrogenase (17β-HSD) (#YJ34923) were obtained from Shanghai Yuanju Biotechnology (China). These kits facilitated the quantification of testosterone, SOD1, SOD2, CAT, CYP17A1, and 17β-HSD levels in both testicular tissue and TM3 cells, following the kit instructions. The absorbance of the samples was measured at a wavelength of 450 nm using the CMax Plus microplate reader (Molecular Devices).

### Western blotting

The homogenized testicular tissue and collected TM3 cells were lysed with RIPA lysis buffer containing protease inhibitor on ice, followed by centrifugation at 12,000 rpm for 20 min at 4°C to obtain the supernatant. The protein concentration was measured using a BCA protein assay kit (P0012S, Beyotime). Equal amounts of protein were separated by 8%-12% SDS-PAGE (P0012A, Beyotime), and the proteins were transferred to a PVDF membrane (IPVH00010, Millipore), which was then blocked with 5% non-fat dry milk for 1 h. The PVDF membrane was incubated overnight at 4°C with rabbit-derived primary antibodies against GRP78 (A23453, ABclonal), GRP94 (A26311, ABclonal), p-PERK (AP1501, ABclonal), PERK (A18196, ABclonal), p-EIF2α (AP0692, ABclonal), EIF2α (A21221, ABclonal), CHOP (A20987, ABclonal), cleaved caspase-3 (A27145, ABclonal), total caspase-3 (A90451, Nature Biosciences), SIRT1 (A0230, ABclonal), ATF4 (A26966, NatureBiosciences) and β-actin (AC026, ABclonal). After washing, it was incubated with goat anti-rabbit horseradish peroxidase-conjugated secondary antibody (A0208, Beyotime) at room temperature for 1 h. The membrane was then subjected to chemiluminescent detection using BeyoECL Plus (P0018S, Beyotime), and imaging was performed using ChemiDOC XRS+ imaging systems (Bio-Rad Laboratories, Hercules, CA, USA). The relative protein expression levels were quantified using ImageJ software.

### Statistical methods

Statistical analysis was conducted using SPSS version 26.0. Data are presented as mean ± standard deviation. One-way analysis of variance with LSD post hoc tests was employed for comparisons among multiple groups, with *P* < 0.05 considered statistically significant.

## Results

### MT improves the function of seminiferous tubules, increases sperm viability and density, and alleviates testicular damage caused by BPA in mice

After 30 days of drug treatment, we compared the testicular index of each group of mice, as illustrated in Figure 1a. The testicular index of the BPA group was significantly lower than that of the control group (*P* = 0.004, *N* = 5 per group). No significant difference was observed between the MT group, the BPA + MT group, and the control group (*P* = 0.980, *P* = 0.301). Notably, the testicular index of the BPA + MT group was significantly higher than that of the BPA group (*P* = 0.033). To assess the function of the seminiferous tubules, testes were subjected to HE staining, with results presented in Figure 1b. The seminiferous epithelium in the testes of the BPA group was thinner compared to that of the control group, characterized by a reduced and disorganized number of germ cells, along with partial shedding of the seminiferous epithelium. In contrast, the testes of the control, MT, and BPA + MT groups displayed thicker seminiferous tubule epithelium, with germ cells arranged in an orderly manner, and no shedding was observed.

**Fig. 1:**
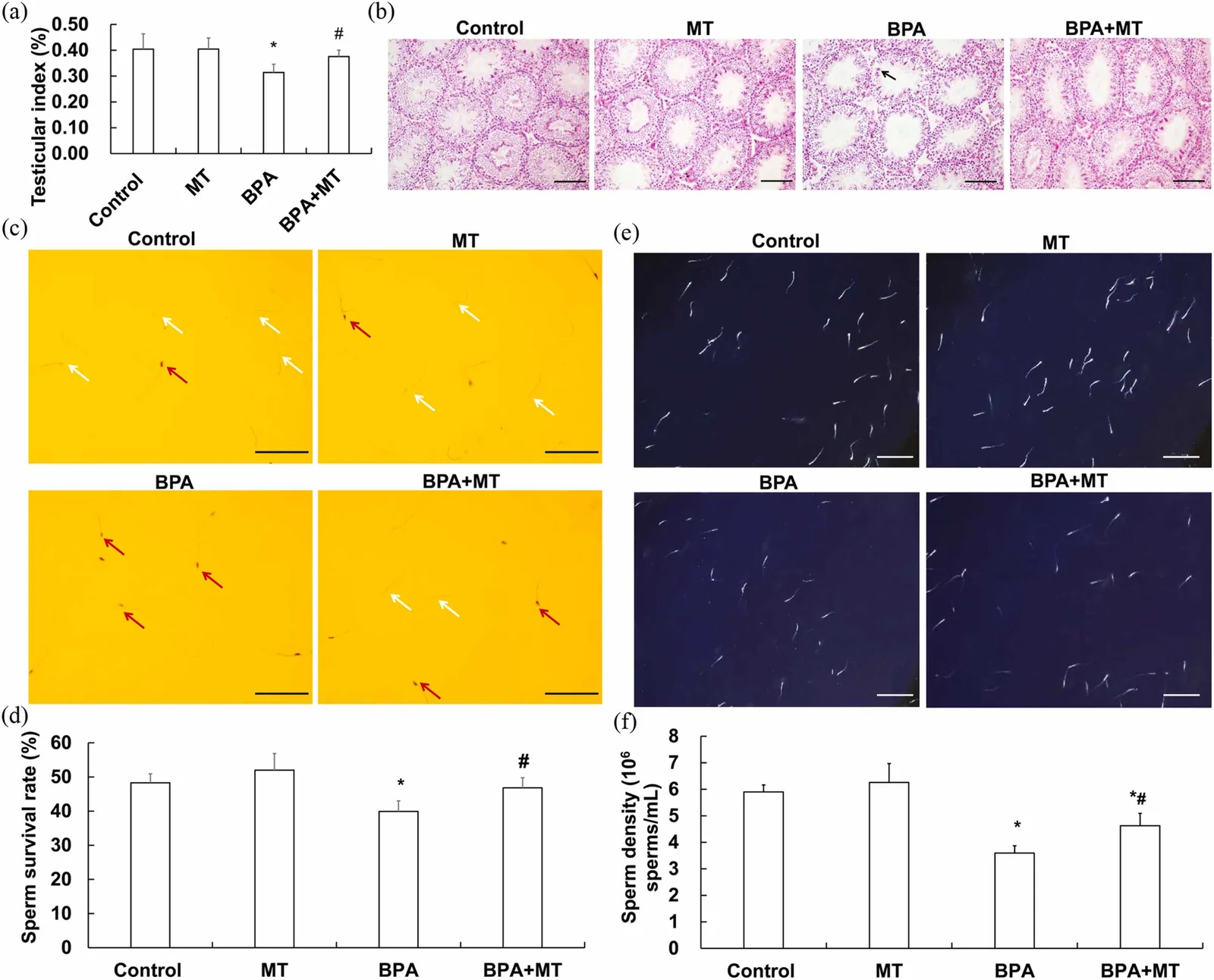
Detection of testicular tissue and epididymal sperm-related indicators in mice. (a) Testicular index of mice (testis weight/body weight ratio) (*N* = 5). (b) HE-stained testicular tissue of mice (scale bar = 100 µm). The arrow indicates the exfoliated seminiferous epithelium. (c) Sperm after eosin staining (white arrows indicate viable sperm, while red arrows indicate dead sperm) (scale bar = 100 µm). (d) Comparison of epididymal sperm survival rate among groups (*N* = 4). (e) Sperm density observed under the microscope (scale bar = 200 µm). (f) Comparison of epididymal sperm density among groups (*N* = 4). Compared with the control group, *P < 0.05; compared with the BPA group, #*P* < 0.05.

To further investigate sperm development in mice, we measured the survival rate and density of sperm from the cauda epididymis. As shown in Figure 1C-F, the sperm survival rate and density in the BPA group were significantly lower than those in the control group (*P* = 0.005, *P* < 0.001, *N* = 4 per group). However, the BPA + MT group had a significantly higher sperm survival rate and density than the BPA group (*P* = 0.016, *P* = 0.009). There were no significant differences between the MT group and the control group (*P* = 0.160, *P* = 0.301). Therefore, MT may not adversely affect normal testicular function and may mitigate the damage induced by BPA, thereby enhancing both the survival rate and density of sperm in mice.

### MT reduces the apoptosis of Leydig cells induced by BPA and enhances their synthesis and secretion of testosterone in mice

TUNEL assay was conducted to detect the apoptosis of Leydig cells in mouse testicular tissue. Cells exhibiting green fluorescence were identified as apoptotic cells with DNA fragmentation. In the testicular tissue of mice, apoptosis primarily occurred in Leydig cells, as illustrated in Figure 2a. Compared to the control group, the number of TUNEL-positive Leydig cells in both the BPA and BPA + MT groups significantly increased (*P* < 0.001, *P* = 0.009; *N* = 5 per group), although the count in the BPA + MT group was markedly lower than that in the BPA group (*P* < 0.001) (Figure 2b). No significant difference was observed in the number of TUNEL-positive Leydig cells between the MT group and the control group (*P* = 0.752), indicating that MT may not adversely affect the function of mouse testes or the apoptosis of Leydig cells; therefore, the MT group was excluded from further experiments.

**Fig. 2:**
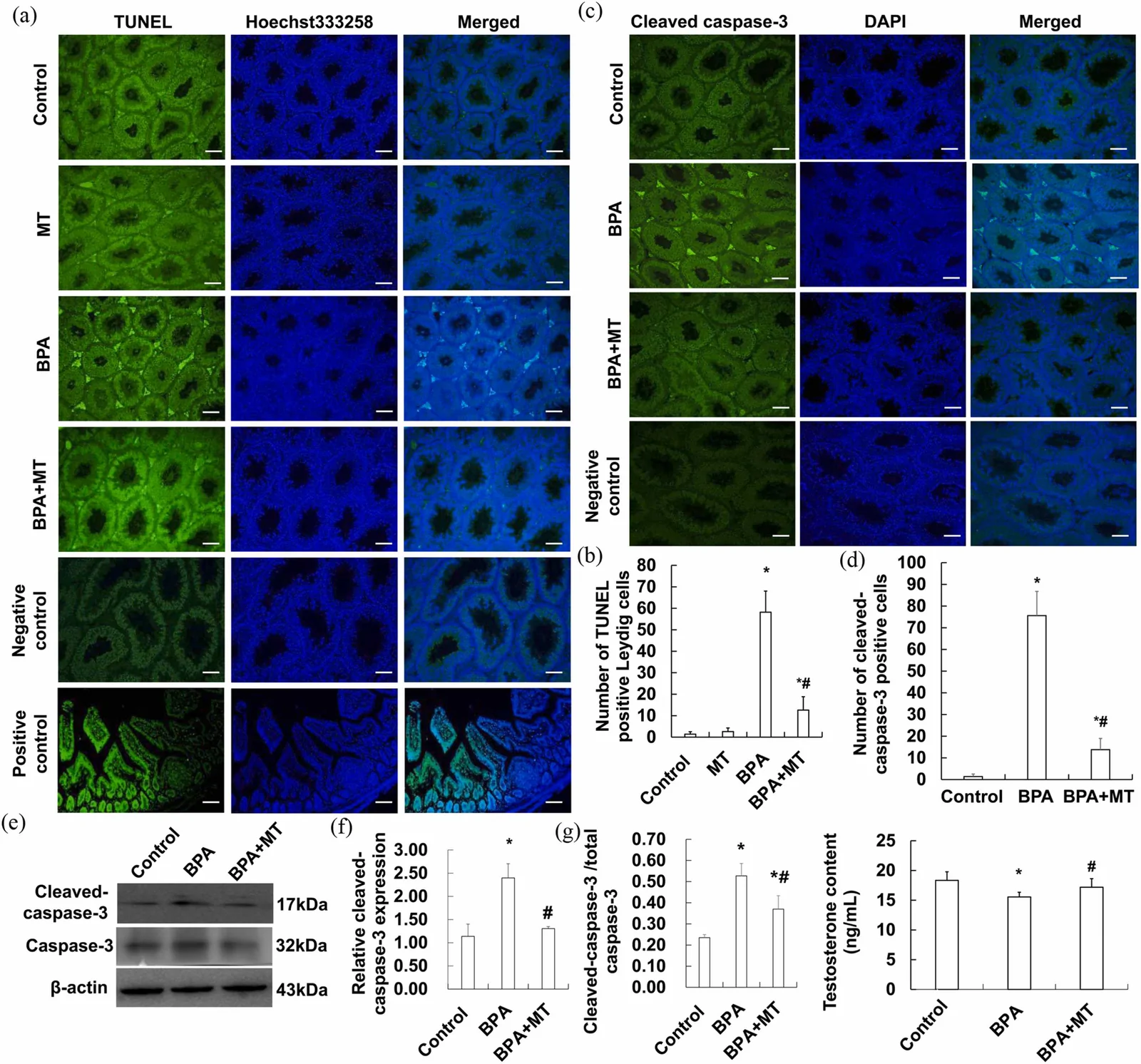
Detection of apoptosis in testicular tissue and testosterone secretion in mice. (a) TUNEL assay detecting apoptosis in mouse testicular tissue. Cells presenting green fluorescence are apoptotic (scale bar = 100 µm). (b) Comparison of TUNEL-positive Leydig cell numbers among groups (*N* = 5). (c) Immunofluorescence detection of cleaved caspase-3 expression in mouse testicular tissue. Cells presenting green fluorescence show positive expression of cleaved caspase-3 (scale bar = 100 µm). (d) Comparison of cleaved caspase-3-positive Leydig cell numbers among groups (*N* = 5). Compared with the control group, *P < 0.05; compared with the BPA group, #*P* < 0.05. (e) Western blotting detection of cleaved caspase-3 and total caspase-3 expression in testicular cells. (f) Relative expression of cleaved caspase-3 protein in testicular cells among groups (*N* = 3). (g) The ratio of cleaved caspase-3 to total caspase-3 in testicular cells among groups (*N* = 3). (h) ELISA detection of testosterone levels in testicular tissue (*N* = 6). Compared with the control group, *P < 0.05; compared with the BPA group, #*P* < 0.05.

Immunofluorescence staining and western blotting analyses detected the expression of cleaved caspase-3 protein in mouse testicular tissue. As shown in Figure 2c, cleaved caspase-3 was predominantly expressed in Leydig cells. The number of cleaved caspase-3-positive Leydig cells (Figure 2d) (*P* = 0.001, *N* = 5 per group) and the protein expression of cleaved caspase-3 (Figure 2e and f) (*P* = 0.001, *N* = 3 per group) in the BPA group were significantly higher than those in the control group. However, both the quantity and expression level in the BPA + MT group were significantly lower than those in the BPA group (*P* < 0.001; *P* = 0.009), with no significant difference compared to the control group (*P* = 0.118; *P* = 0.412). To further confirm caspase-3 activation, we detected total caspase-3 and calculated the ratio of cleaved caspase-3 to total caspase-3. As shown in Figure 2e and g, the cleaved caspase-3/total caspase-3 ratio was significantly increased in the BPA group compared with the control group (*P* < 0.001, *N* = 3) and was significantly reduced in the BPA + MT group compared with the BPA group (*P* = 0.009, *N* = 3). Thus, MT effectively diminished the expression of cleaved caspase-3 in Leydig cells of BPA-treated mice, consistent with its inhibitory role in apoptosis.

To assess the impact of BPA and MT on testosterone synthesis and secretion, testosterone levels in mouse testicular tissue were measured by using ELISA. The results revealed that the testosterone levels in the testicular tissue of mice in the BPA group were significantly lower than those in the control group (*P* = 0.002, *N* = 6 per group) (Figure 2h). In contrast, testosterone levels in the BPA + MT group were significantly higher than those in the BPA group (*P* = 0.039), with no significant difference compared to the control group (*P* = 0.137).

Collectively, MT inhibited BPA-induced Leydig cell apoptosis and improved testosterone synthesis and secretion in these cells.

### MT mitigates MBP-induced TM3 cell apoptosis and ERS suppression

To further investigate the effects of MT and the metabolite MBP of BPA on Leydig cells *in vitro*, we introduced MT and MBP into the culture medium of TM3 cells and evaluated their survival and apoptosis. The CCK-8 assay indicated that the survival rate of TM3 cells in both the MBP group and the MBP + MT group was significantly lower than that of the control group (both *P* < 0.001, *N* = 8 per group); however, the survival rate of TM3 cells in the MBP + MT group was significantly greater than that in the MBP group (Figure 3a) (*P* < 0.001). Flow cytometry was employed to assess TM3 cell apoptosis. As shown in Figure 3b and c, the apoptosis rate of TM3 cells in the MBP group and the MBP + MT group was significantly higher than that of the control group (*P* < 0.001, *P* = 0.004; *N* = 6 per group), while the survival rate of TM3 cells in the MBP + MT group remained significantly lower than that in the MBP group (*P* < 0.001). For subsequent experiments, TM3 cells were treated with 13 μM MBP for 24 h. Pre-treatment with MT before MBP exposure may enhance the cell viability and mitigate MBP-induced apoptosis of TM3 cells.

**Fig. 3:**
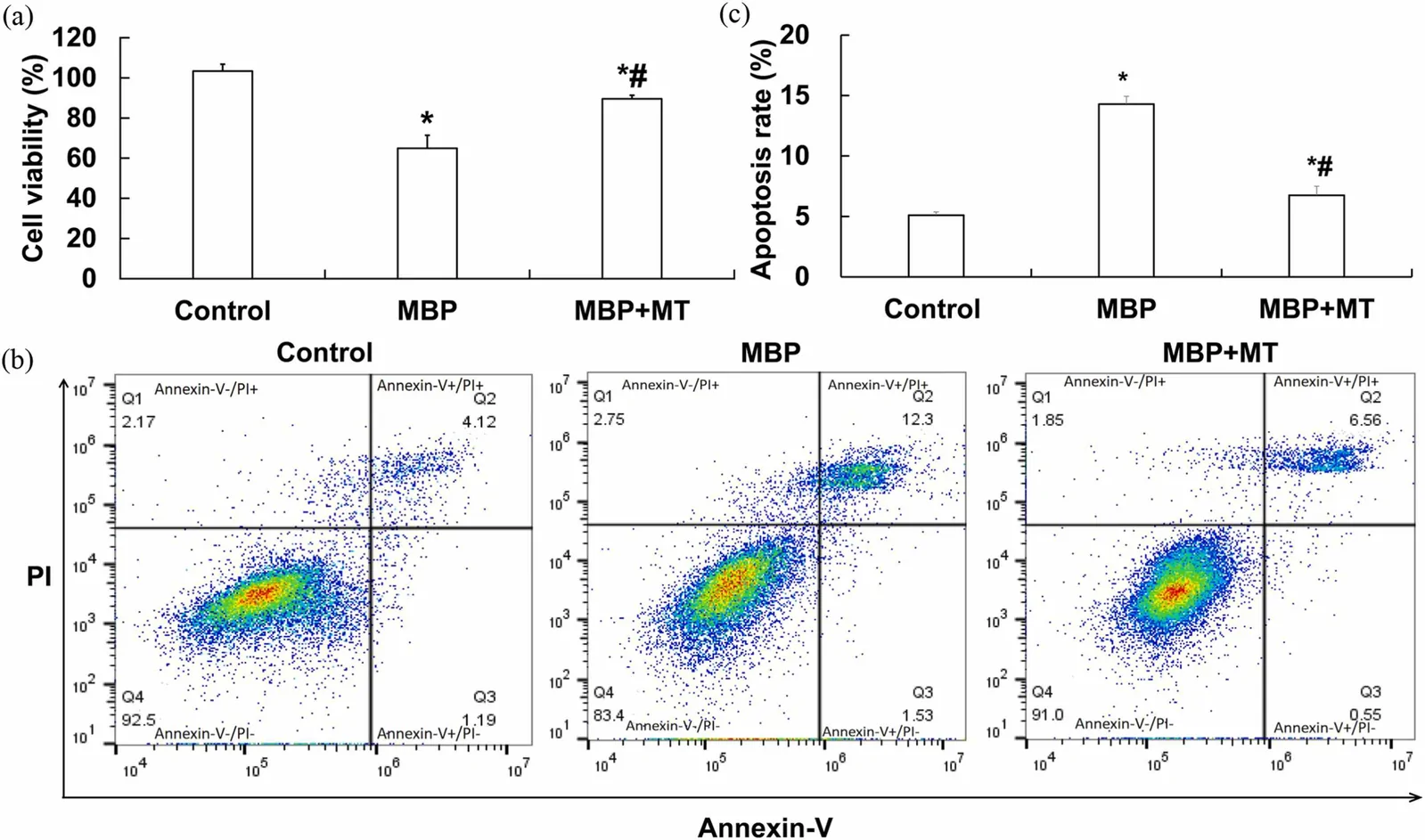
Effects of MBP and MT on TM3 cell viability. (a) Viability of TM3 cells after treatment with MT and MBP (*N* = 8). *Compared with the control group, *P* < 0.05; #Compared with the MBP group, *P* < 0.05. (B) Detection of cell apoptosis by flow cytometry. (C) Comparison of apoptosis rates among TM3 cells (*N* = 6). *Compared with the control group, *P* < 0.05; #Compared with the MBP group, *P* < 0.05.

Moreover, the TUNEL assay was performed to analyze the effects of MBP and MT on DNA fragmentation in TM3 cells. Compared to the control group, the proportion of TUNEL-positive cells significantly increased in both the MBP and MBP + MT groups (*P* < 0.001, *P* = 0.016; *N* = 3 per group); however, the rate in the MBP + MT group was significantly lower than that in the MBP group (*P* < 0.001) (Figure 4a and b). TM3 was then intervened with the ERS activator TG. TUNEL staining revealed that the proportion of TUNEL-positive TM3 cells in the MBP + MT and TG + MT groups was significantly lower than that in the MBP and TG groups (both *P* < 0.001) (Figure 4a and c). This result indicates that MT alleviates the apoptosis in TM3 cells induced by MBP and TG, which may be achieved through inhibiting the ERS.

**Fig. 4:**
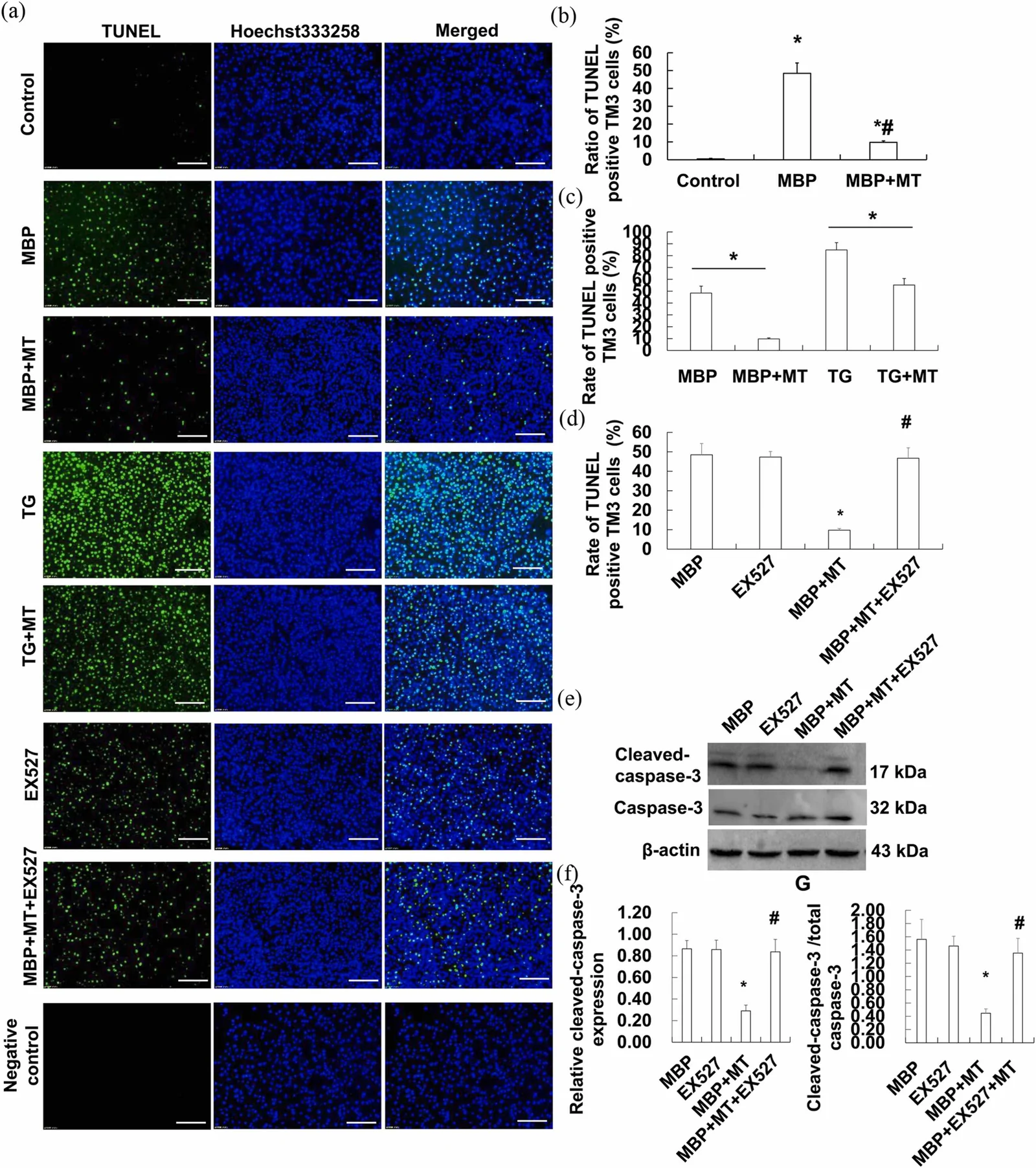
TUNEL detection of apoptosis in TM3 Cells. (a) TUNEL assay detecting apoptosis in mouse TM3 cells. Cells presenting green fluorescence are apoptotic (scale bar = 150 μm). (b) Comparison of the ratio of TUNEL-positive TM3 cells among groups (*N* = 3). Compared with the control group, *P < 0.05; compared with the MBP group, #*P* < 0.05. (c) Comparison of the ratio of TUNEL-positive TM3 cells among groups (*N* = 3). Compared with the MBP or TG group, *P < 0.05. (d) Comparison of the ratio of TUNEL-positive TM3 cells among groups (*N* = 3). Compared with the MBP group, *P < 0.05; compared with the MBP+MT group, #*P* < 0.05. (e) Representative western blotting images of cleaved caspase-3 and total caspase-3 expression in TM3 cells. (f) Relative expression of cleaved caspase-3 protein among groups (*N* = 3). (g) The ratio of cleaved caspase-3 to total caspase-3 in testicular cells among groups (*N* = 3). Compared with the MBP group, *P < 0.05; compared with MBP+MT group, #*P* < 0.05.

Additionally, we analyzed the role of SIRT1 in the alleviation of MBP-triggered apoptosis by MT in TM3 cells after treatment with the SIRT1 inhibitor EX527. TUNEL assay and western blotting analysis revealed that the proportion of TUNEL-positive TM3 cells and the expression of cleaved caspase-3 in the EX527 group showed no significant difference compared to the MBP group (*P* = 0.561, *P* = 0.952; *N* = 3 per group) (Figure 4a, d-f). However, in the MBP + MT group, these levels were significantly lower than those in the MBP group (*P* < 0.001) (Figure 4a, d-f). Notably, this effect was reversed after treatment with EX527, as shown by significantly increased TUNEL-positive cell proportion and cleaved caspase-3 expression in the MBP + MT + EX527 group than in the MBP + MT group (*P* < 0.001) (Figure 4a, d-f). Consistently, the ratio of cleaved caspase-3 to total caspase-3 showed the same trend: it was not significantly different between the EX527 and MBP groups (*P* = 0.833), was significantly reduced in the MBP + MT group compared with the MBP group (*P* < 0.001), and was significantly elevated again in the MBP + MT + EX527 group compared with the MBP + MT group (*P* < 0.001) (Figure 4e and g). These results indicate that MT may inhibit apoptosis in TM3 cells of the testes induced by MBP, which may be associated with SIRT1.

## MT mitigates the reduction in testosterone secretion

To further explore the impact of MBP and MT on Leydig cell functionality, ELISA was used to measure testosterone levels along with steroidogenic enzymes CYP17A1 and 17β-HSD in TM3 cells. Notably, the levels of testosterone, CYP17A1, and 17β-HSD in both the MBP and MBP + MT groups were significantly lower than in the control group (*P* = 0.045, *P* = 0.026, and *P* = 0.002, respectively; *N* = 9 per group), while the levels in the MBP + MT group were significantly elevated compared to the MBP group (*P* < 0.001, *P* < 0.001, and *P* = 0.001, respectively) (Figure 5a-c). Thus, MT alleviated DNA fragmentation in TM3 cells induced by MBP, enhanced the expression of steroidogenic enzymes, and promoted testosterone secretion.

**Fig. 5:**
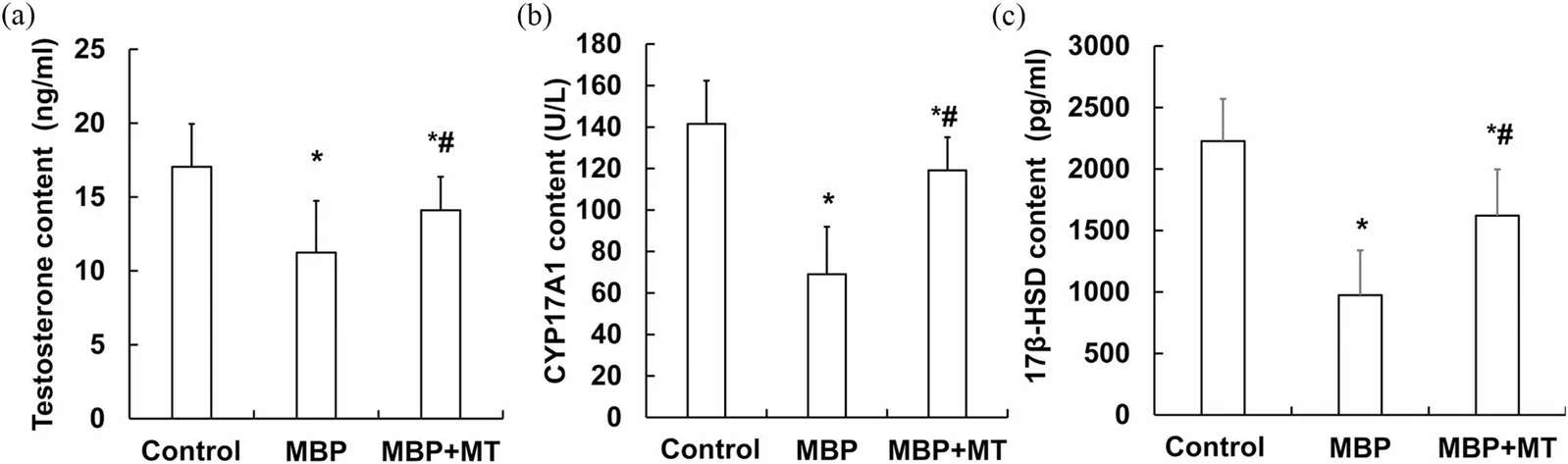
ELISA detection of testosterone secretion and steroidogenic enzyme levels in TM3 Cells. (A) Testosterone levels in TM3 cells (*N* = 9). (B) CYP17A1 levels in TM3 cells (*N* = 9). (C) 17β-HSD levels in TM3 cells (*N* = 9). *Compared with the control group, *P* < 0.05; #Compared with the MBP group, *P* < 0.05.

### MT alleviates the oxidative stress in TM3 cells induced by MBP

To understand the mechanisms by which MBP induces DNA fragmentation and cell death in TM3 cells and the avenues through which MT exerts its effects, we assessed the level of oxidative stress in TM3 cells. The levels of ROS in TM3 cells were measured using DCFH-DA probes, as illustrated in Figure 6a. The ROS levels in the MBP and MBP + MT groups of TM3 cells were significantly higher than those in the control group (both *P* < 0.001; *N* = 3 per group), while those in the MBP + MT group of TM3 cells were significantly lower than those in the MBP group (P = 0.001) (Figure 6b). Additionally, the levels of antioxidant enzymes CAT, SOD1, and SOD2 in TM3 cells were measured using ELISA. It was found that their levels in both the MBP and MBP + MT groups were significantly lower than those in the control group (all *P* < 0.001 in the MBP group; *P* = 0.042, *P* < 0.001, and *P* < 0.001 in the MBP + MT group, respectively; *N* = 9 per group) (Figure 6c-e). However, their levels in the MBP + MT group were significantly higher than those in the MBP group (*P* = 0.001, *P* < 0.001, and *P* < 0.001, respectively). Therefore, MT enhanced the expression of antioxidant enzymes and reduced ROS levels, thereby alleviating the oxidative stress induced by MBP in TM3 cells.

**Fig. 6:**
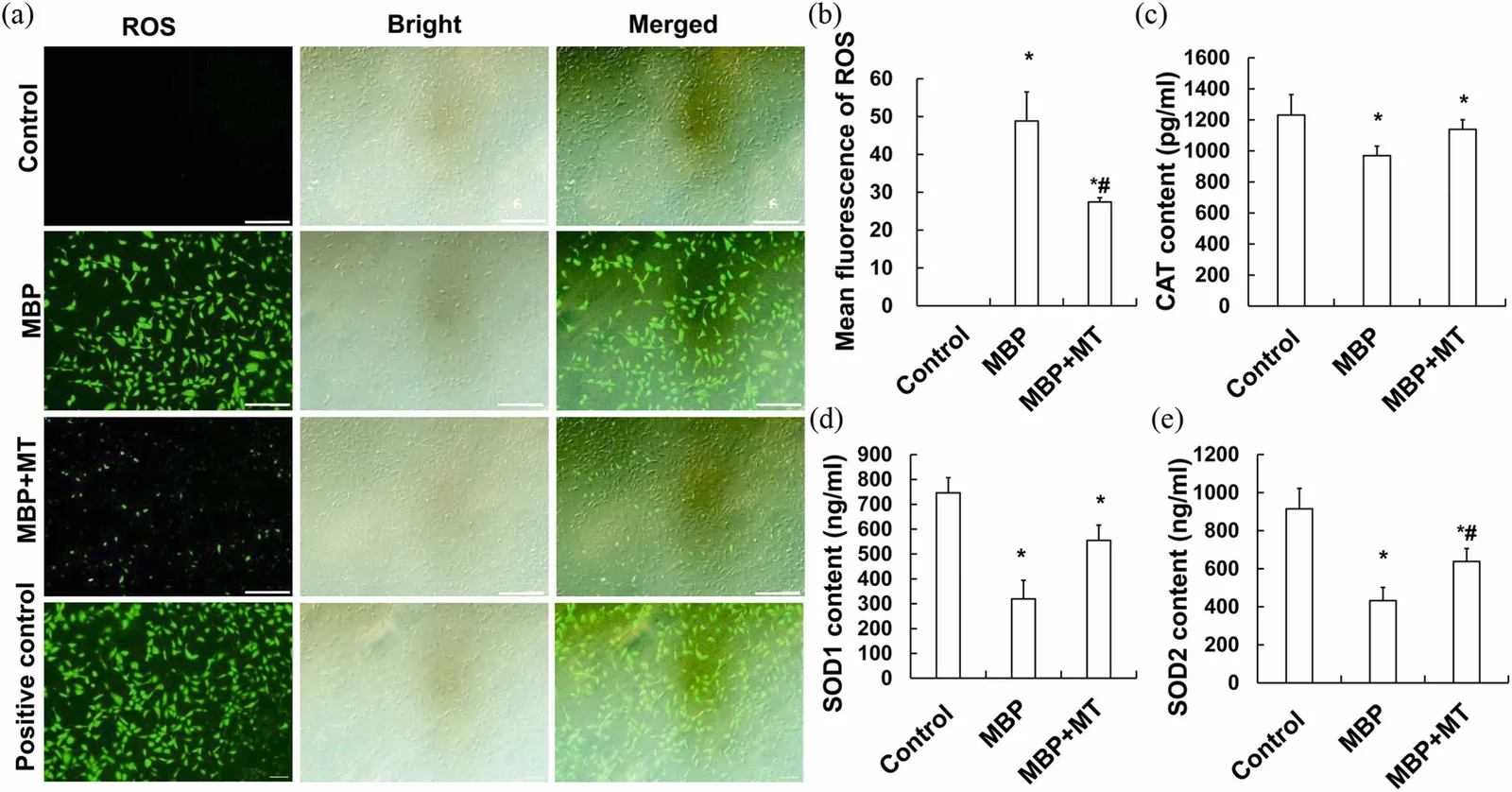
Levels of ROS and antioxidant enzymes in TM3 cells. (A) ROS staining with DCFH-DA in TM3 cells. Scale bar = 25 μm. (B) Average fluorescence intensity of ROS (*N* = 3). (C) ELISA detection of CAT levels in TM3 cells (*N* = 9). (D) ELISA detection of SOD1 levels in TM3 cells (*N* = 9). (E) ELISA detection of SOD2 levels in TM3 cells (*N* = 9). Compared with the control group, *P < 0.05; compared with the MBP group, #*P* < 0.05.

### MT attenuates the BPA-induced ERS in testicular tissues

Oxidative stress can disrupt the redox state of the ER, leading to ERS. This study used immunofluorescence staining and western blotting to investigate the effects of BPA and MT on the expression of ERS markers GRP94 and GRP78, as well as the activation of the PERK signaling pathway in mouse testicular tissue. Immunofluorescence staining demonstrated that GRP94, GRP78, and CHOP were primarily expressed in Leydig cells of the testes (Figure 7A, C, and E). Both immunofluorescence staining and western blotting analysis revealed that in the BPA group, the expression levels of GRP94, GRP78, and CHOP were significantly higher than those in the control group (all *P* < 0.001, *N* = 5 per group for Figure 7b, d, and f; GRP94: *P* < 0.001, GRP78: *P* = 0.020, CHOP: *P* < 0.001, *N* = 3 per group for Figure 8a-d). However, this effect of BPA was attenuated by MT, as evidenced by the reduced levels of GRP94, GRP78, and CHOP in the BPA + MT group (all *P* < 0.001 in Figure 7b, d, and f; *P* = 0.001, *P* = 0.054, and *P* = 0.001 in Figure 8a-d, respectively). Additionally, western blotting analysis showed that BPA significantly increased the expression levels of p-PERK/PERK and p-EIF2α/EIF2α in the PERK signaling pathway compared to the control group (both *P* < 0.001), whereas in the BPA + MT group, the levels of these proteins were significantly reduced compared to the BPA group (*P* = 0.024, *P* = 0.002) (Figure 8e-h). These results indicate that BPA-induced ERS in testicular tissue could be alleviated by MT.

**Fig. 7:**
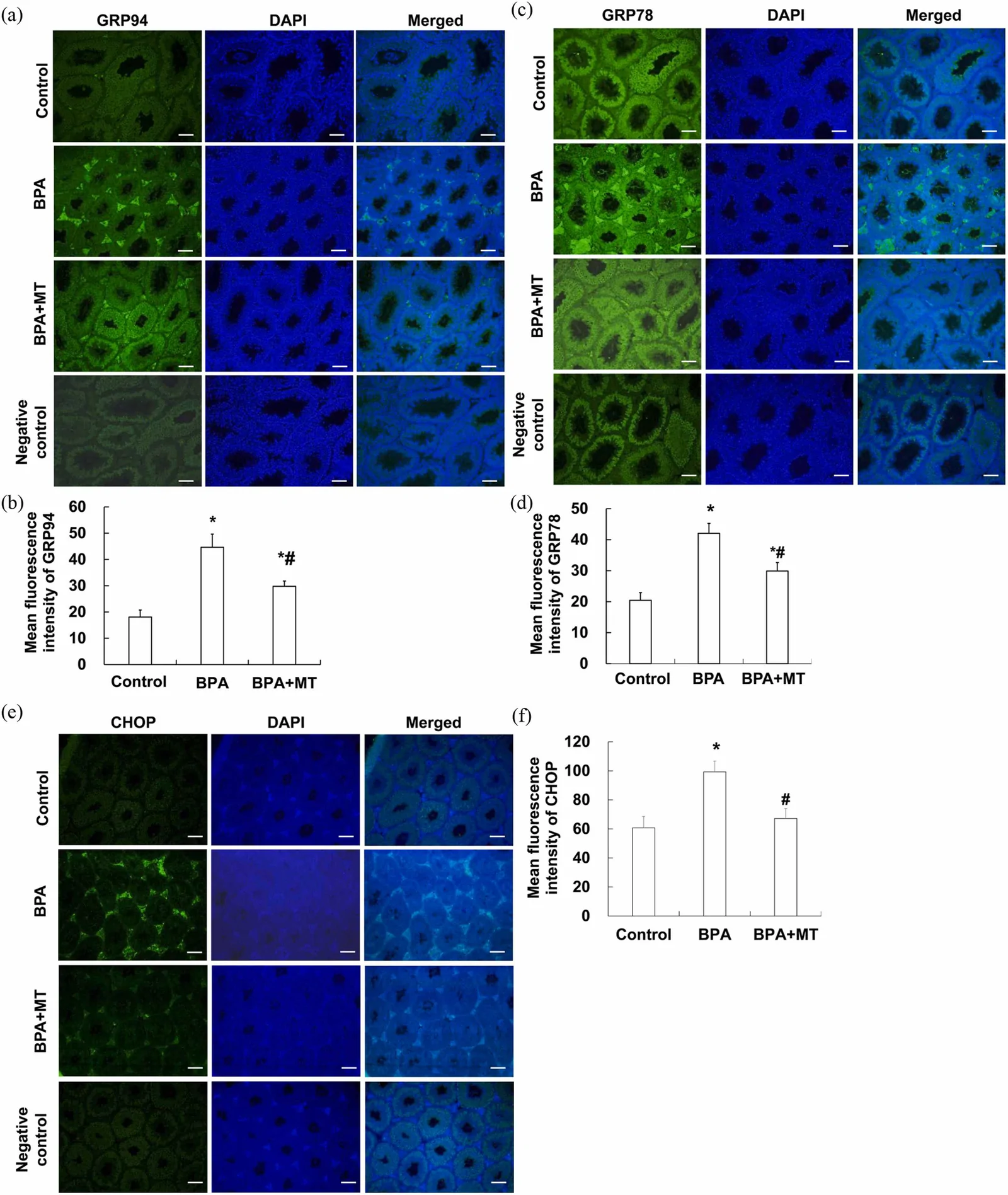
Detection of GRP94 and GRP78 expression in mouse testicular tissue using immunofluorescence. (a) Immunofluorescence detection of GRP94 expression in mouse testicular tissue. GRP94-positive expression presents green fluorescence (scale bar = 100 μm). (b) Average fluorescence intensity of GRP94 (*N* = 5). (c) Immunofluorescence detection of GRP78 expression in mouse testicular tissue. GRP78-positive expression displays green fluorescence (scale bar = 100 μm). (d) Average fluorescence intensity of GRP78 (*N* = 5). (e) Immunofluorescence detection of CHOP expression in mouse testicular tissue. CHOP-positive expression displays green fluorescence (scale bar = 100 μm). (f) Average fluorescence intensity of CHOP (*N* = 5). Compared with the control group, *P < 0.05; compared with the BPA group, #*P* < 0.05.

**Fig. 8:**
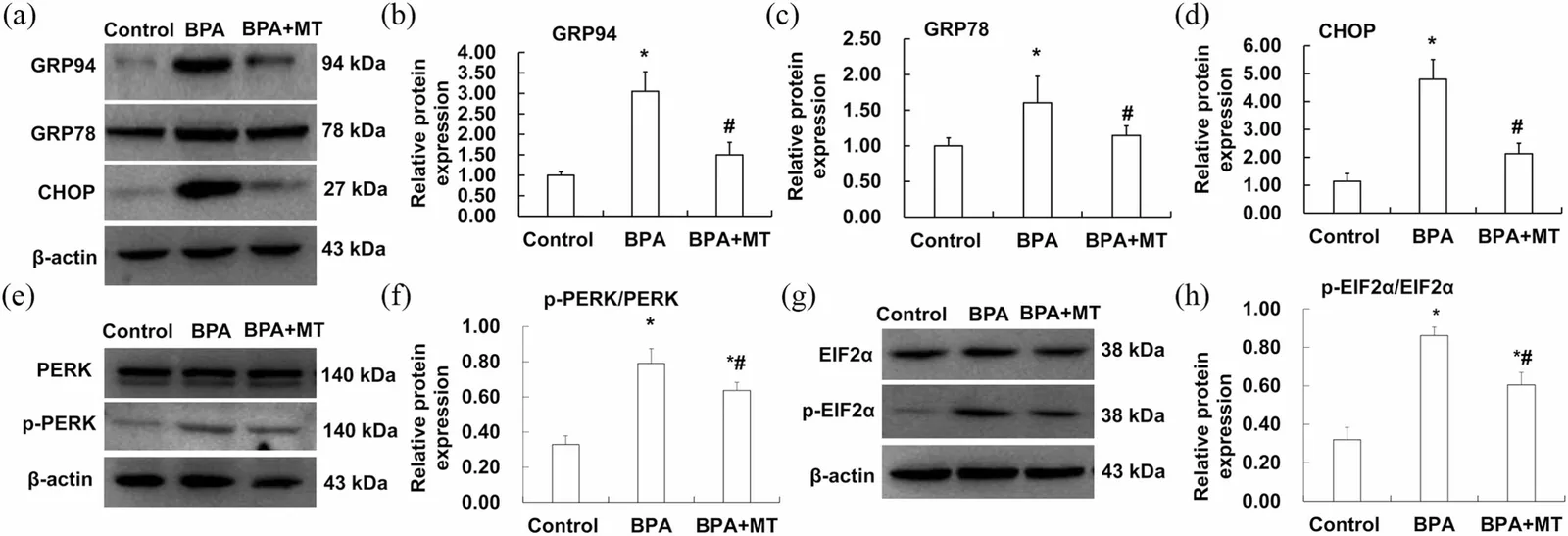
Western blotting for detection of ERS-related protein expression in testicular tissue. (a) Representative western blotting images of GRP94, GRP78, and CHOP proteins in testicular tissue. Relative expression of GRP94 (b), GRP78 (c), and CHOP (d) in testicular tissue (*N* = 3). (e) Representative western blotting images of PERK and p-PERK protein expression in testicular tissue. (f) Ratio of p-PERK to PERK in testicular tissue (*N* = 3). (g) Representative western blotting images of EIF2α and p-EIF2α protein expression in testicular tissue. (h) Ratio of p-EIF2α to EIF2α in testicular tissue (*N* = 3). Compared with the control group, *P < 0.05; compared with the BPA group, #*P* < 0.05.

### MT inhibits ERS in TM3 cells exposed to MBP

To further investigate the effects of MBP and MT on ERS in TM3 cells, we measured the expression of ERS markers and the activation of the PERK signaling pathway. Western blot analysis revealed that the expression levels of GRP94 and GRP78, as well as the PERK pathway-associated proteins p-PERK/PERK, p-EIF2α/EIF2α, ATF4, and CHOP, were significantly elevated in both the MBP and TG groups compared to the control group (all *P* < 0.001, except for CHOP in the MBP group, *P* = 0.005; all *P* < 0.001 in the TG group; *N* = 3 per group) (Figure 9a-f). In contrast, treatment with MT significantly reduced the expression of these proteins in the MBP + MT and TG + MT groups compared to the MBP (*P* = 0.003, *P* = 0.002, *P* = 0.002, *P* = 0.001, *P* = 0.006, and *P* = 0.015, respectively) and TG groups, respectively (*P* < 0.001, *P* = 0.013, *P* = 0.002, *P* < 0.001, *P* = 0.002, and *P* = 0.001, respectively). These findings indicate that MBP, similar to the classical ERS activator TG, induces ERS in TM3 cells and that MT effectively alleviates ERS through inhibition of the PERK signaling pathway.

**Fig. 9:**
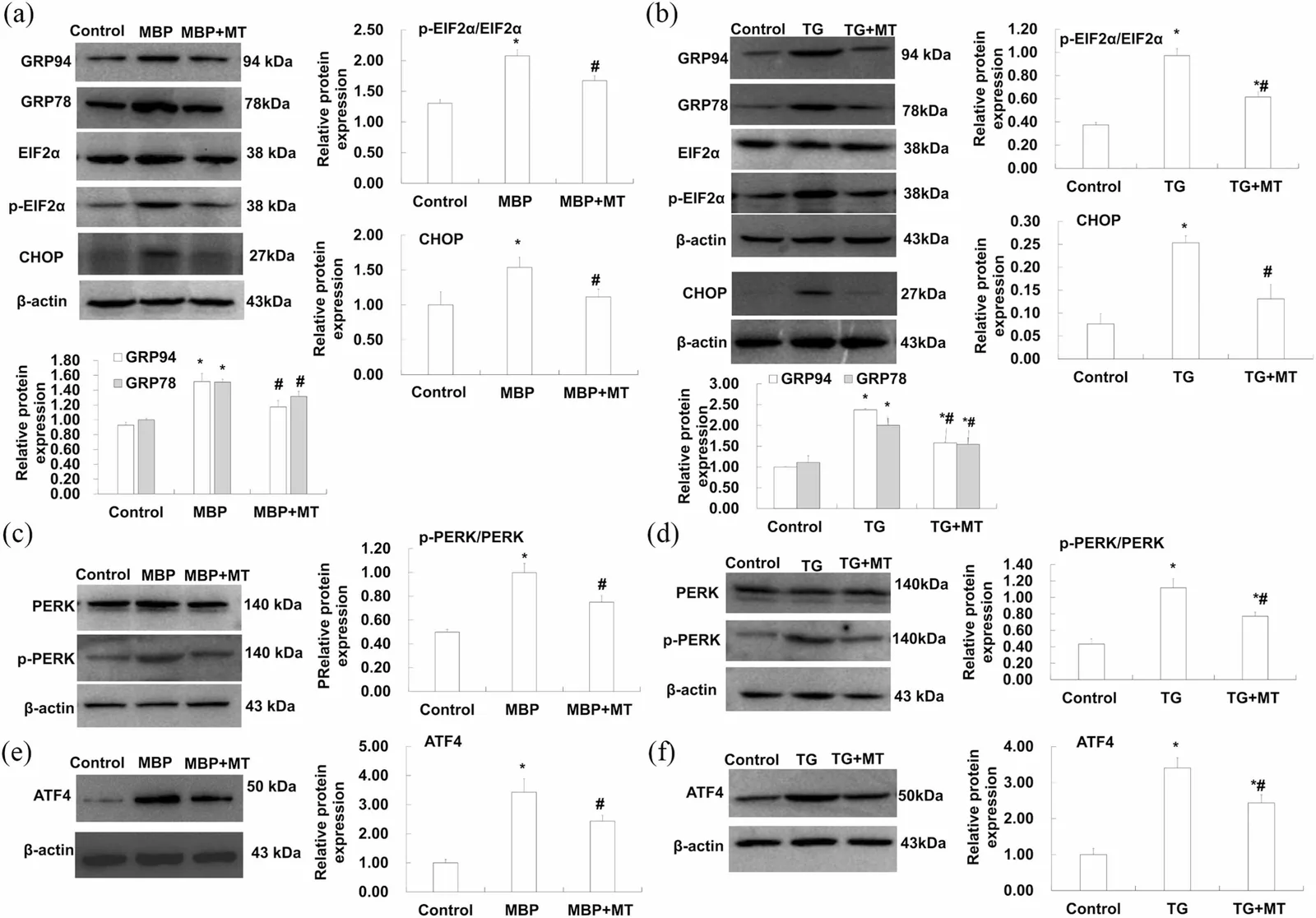
Expression of ERS-related proteins in TM3 Cells. Protein expression was measured with western blotting. (a and b) Representative and quantitative expression of GRP94, GRP78, EIF2α, p-EIF2α, and CHOP in TM3 cells among groups treated with MBP and MT (*N* = 3) (a) and groups treated with TG and MT (*N* = 3) (b). (c and d) Representative expression of p-PERK and PERK as well as the p-PERK/PERK ratio in TM3 cells among groups treated with MBP and MT (*N* = 3) (c) and groups treated with TG and MT (*N* = 3) (D). (e and f) Representative and quantitative expression of ATF4 in TM3 cells among groups treated with MBP and MT (*N* = 3) (e) and groups treated with TG and MT (*N* = 3) (f). Compared with the control group, *P < 0.05; compared with the MBP or TG group, #*P* < 0.05.

### MT induces SIRT1 activation in TM3 cells: Evidence from expression, subcellular localization, and enzymatic activity

SIRT1 is an important factor regulating ERS.^21^ To investigate whether MT activates SIRT1 in TM3 cells, we first assessed SIRT1 expression after different durations of MT treatment. As shown in Figure 10a and b, a 6-h treatment with MT significantly increased SIRT1 expression in TM3 cells (all *P* < 0.001; *N* = 3 per group). Therefore, a 6-h MT pre-treatment was used in subsequent experiments.

**Fig. 10:**
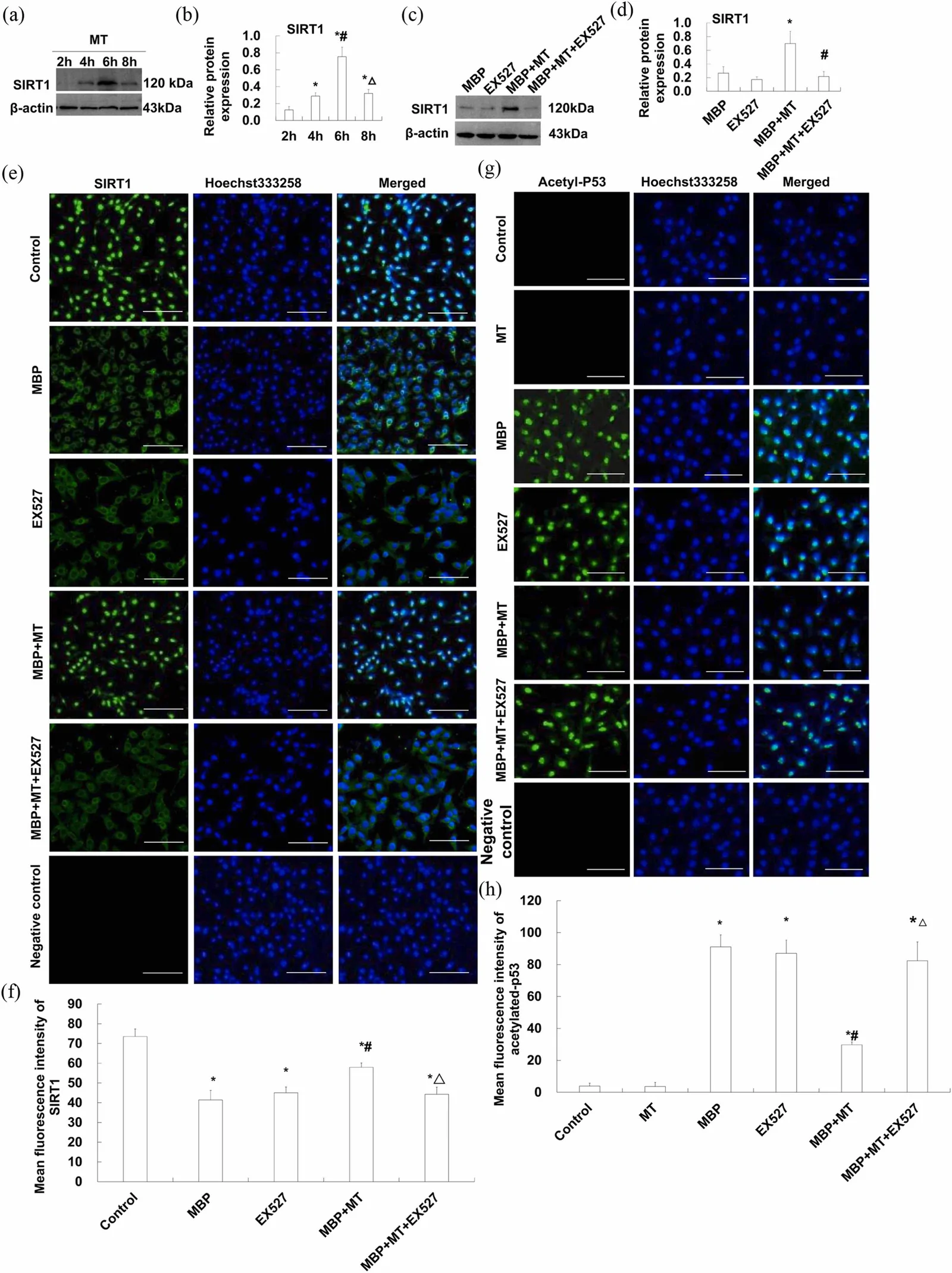
MT activates SIRT1 and enhances its nuclear localization and deacetylase activity in TM3 cells. (a) Representative western blotting images of SIRT1 expression in TM3 cells treated with melatonin for different durations (2, 4, 6, 8 h). (b) Quantitative analysis of SIRT1 protein levels relative to β-actin (*N* = 3). (c) Representative western blotting images of SIRT1 expression in TM3 cells treated with MBP, MT, and/or EX527. (d) Quantitative analysis of SIRT1 protein levels relative to β-actin (*N* = 3). For (b) and (d), Compared with the MT 2 h group or MBP group, *P < 0.05; compared with the MT 4 h group or MBP + MT group, #*P* < 0.05; compared with the MT 6 h group, △P<0.05. (e) Immunofluorescence staining of SIRT1 (green) in TM3 cells under different treatment conditions. Nuclei were counterstained with DAPI (blue). Scale bar = 100 μm. (f) Quantitative analysis of nuclear SIRT1 fluorescence intensity (*N* = 5). (G) Immunofluorescence staining of acetylated-p53 (green) in TM3 cells. (h) Quantitative analysis of acetylated-p53 fluorescence intensity (*N* = 5). For (f) and (h), compared with the control group, *P < 0.05; compared with the MBP group, #*P* < 0.05, compared with the MBP+MT group, △*P* < 0.05.

We further examined total SIRT1 expression in TM3 cells under different treatment conditions. As shown in Figure 10c and d, no significant difference in SIRT1 protein levels was observed between the EX527 group and the MBP group (*P* = 0.326). In contrast, SIRT1 expression in the MBP + MT group was significantly higher than that in the MBP group (*P* = 0.001). This upregulation was abolished by EX527 co-treatment, as SIRT1 expression in the MBP + MT + EX527 group was significantly lower than that in the MBP + MT group (*P* = 0.001) (Figure 10c and d).

Immunofluorescence staining was performed to examine the subcellular localization of SIRT1. As shown in Figure 10e and f, SIRT1 was predominantly localized in the nucleus of control cells. However, both MBP and EX527 treatments induced significant nuclear exclusion of SIRT1, with the protein primarily redistributed to the cytoplasm (both *P* < 0.001, *N* = 5 per group). MT treatment effectively reversed MBP-induced nuclear exclusion, restoring SIRT1 to the nucleus (*P* < 0.001). Notably, this effect of MT was abolished by EX527 co-treatment, as SIRT1 remained predominantly cytoplasmic in the MBP + MT + EX527 group, with no significant difference compared to the MBP group (*P* = 0.228).

To evaluate whether the nuclear-localized SIRT1 was functionally active, we examined the acetylation level of its downstream target p53 (Lys382). As shown in Figure 10g and h, acetylated-p53 levels were significantly elevated in MBP-treated cells compared to the control group (*P* < 0.001), indicating reduced SIRT1 activity. EX527 treatment alone similarly elevated acetylated-p53 levels, with no significant difference from the MBP group (*P* > 0.999), confirming that 10 μM EX527 effectively inhibits SIRT1 deacetylase activity in TM3 cells. In contrast, no significant difference in p53 acetylation was observed between the MT group and the control group (*P* > 0.999). Notably, MT treatment significantly decreased p53 acetylation in MBP-treated cells (*P* < 0.001), demonstrating that MT restores SIRT1 enzymatic activity under MBP-induced stress conditions. This effect of MT was reversed by EX527 co-treatment, as evidenced by significantly increased acetylated-p53 levels in the MBP + MT + EX527 group compared to the MBP + MT group (*P* = 0.007).

Collectively, these results demonstrate that MT induces SIRT1 activation in TM3 cells through multiple mechanisms: upregulating total protein expression, promoting nuclear retention, and enhancing deacetylase activity. All of these effects were attenuated by the SIRT1 inhibitor EX527, confirming the specificity of SIRT1 involvement.

### MT alleviates MBP-induced ERS in TM3 cells through SIRT1 activation

To further evaluate the role of SIRT1 in MT-mediated ERS alleviation, TM3 cells were treated with the SIRT1 inhibitor EX527. As shown in Figure 11a-c, no significant differences were observed in the expression levels of GRP94, GRP78, p-PERK/PERK, p-EIF2α/EIF2α, ATF4, or CHOP between the EX527 group and the MBP group (*P* = 0.489, *P* = 0.755, *P* = 0.564, *P* = 0.886, and *P* = 0.945, respectively; *N* = 3 per group). However, compared to the MBP group, the MBP + MT group exhibited significantly lower expression of these proteins (*P* = 0.001, *P* = 0.001, *P* = 0.006, *P* = 0.030, and *P* < 0.001, respectively). Notably, this suppressive effect of MT was reversed by EX527 treatment, as evidenced by significantly increased protein expression in the MBP + MT + EX527 group compared to the MBP + MT group (*P* = 0.009, *P* = 0.001, *P* = 0.001, *P* = 0.014, and *P* < 0.001, respectively). These results suggest that MT alleviates MBP-induced ERS in TM3 cells at least partially through activation of the SIRT1 pathway.

**Fig. 11:**
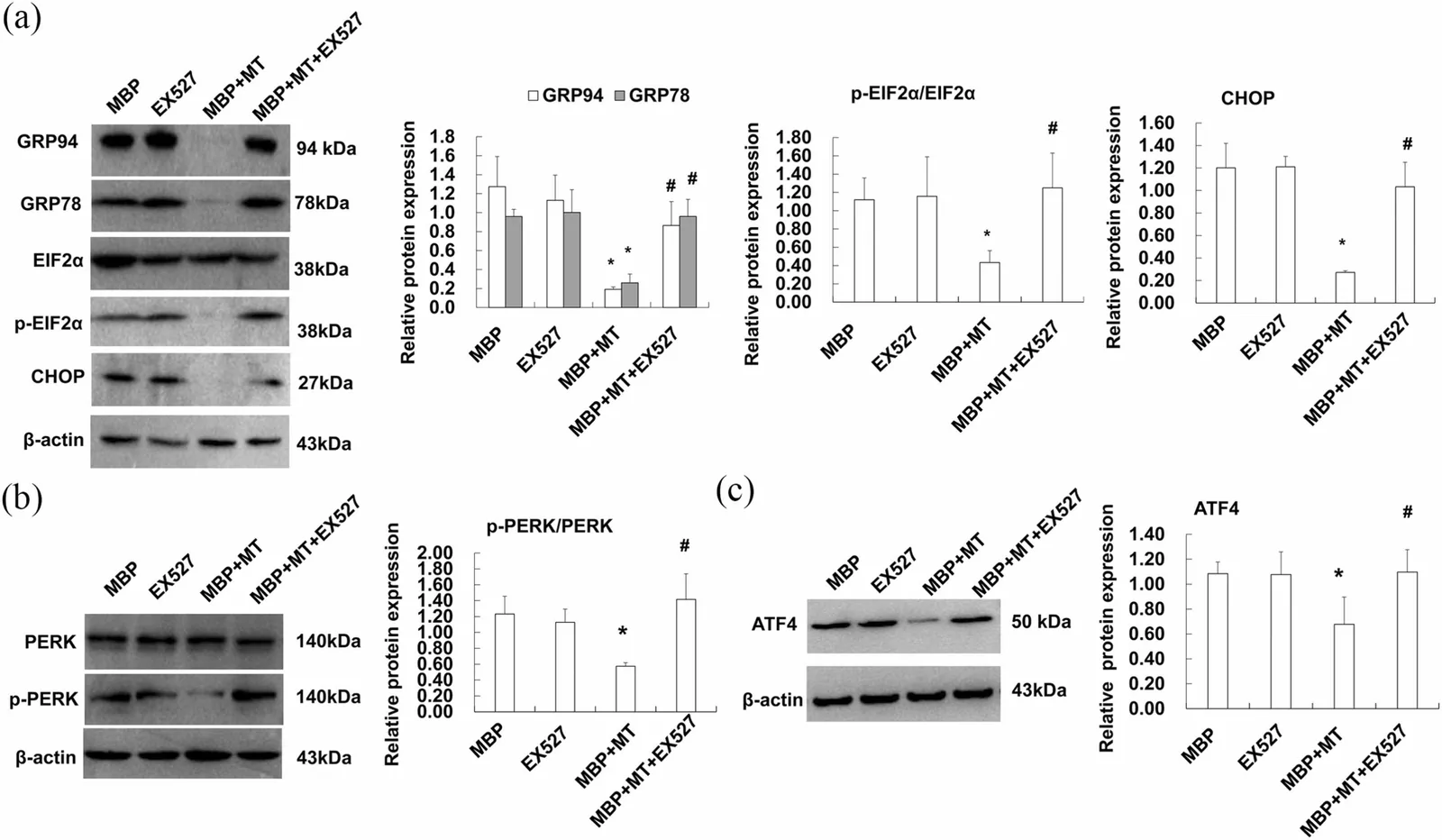
Expression of ERS-related proteins in mouse TM3 Cells. Protein expression was measured with western blotting. (a) Representative and quantitative expression of GRP94, GRP78, EIF2α, p-EIF2α, and CHOP in TM3 cells among groups (*N* = 3). (b) Representative expression of p-PERK and PERK as well as the p-PERK/PERK ratio in TM3 cells among groups (*N* = 3). (c) Representative and quantitative expression of ATF4 in TM3 cells among groups (*N* = 3). Compared with the MBP group, *P < 0.05; compared with the MBP + MT group, #*P* < 0.05.

## Discussion

BPA, as an environmental pollutant, negatively impacts the male reproductive system, particularly affecting spermatogenesis and testosterone production.^22^ Our previous research has revealed that MT, as an antioxidant, could alleviate BPA-induced damage to the testes.^5^ However, the specific mechanisms remain unclear. In this study, we found through *in vivo* and *in vitro* experiments that MT significantly reduced apoptosis in Leydig cells induced by BPA and its metabolite MBP while enhancing testosterone synthesis. Furthermore, we clarified that these protective effects of MT were closely related to its activation of SIRT1 and inhibition of the PERK/EIF2α/ATF4/CHOP pathway, which alleviated oxidative stress and ERS in Leydig cells. These results suggest that MT may serve as a novel therapeutic strategy to protect male reproductive health from the harmful effects of endocrine disruptors.

BPA is metabolized in the liver, leading to the production of various metabolites, among which MBP is a primary compound. Since MBP exhibits stronger cytotoxicity and estrogenic activity than BPA,^7^ we used MBP as the direct toxicant in the *in vitro* cell experiments to more closely mimic the *in vivo* exposure scenario, thereby bridging the *in vivo* BPA data with the *in vitro* MBP data. MBP has been shown to increase the production of ROS in nerve cells, thereby disrupting the balance between ROS and the cellular antioxidant defenses, which triggers oxidative stress.^23^ This oxidative stress can result in multiple pathological conditions, including cellular damage, inflammation, and apoptosis.^24^ Furthermore, cellular oxidative stress can negatively impact organelles, particularly the ER, leading to ERS and interfering with the processing and handling of proteins. Consequently, this results in the accumulation of misfolded proteins, the induction of the UPR, and an elevation in the expression of UPR markers. Long-term ERS can activate apoptotic pathways, further exacerbating cellular damage and ultimately resulting in cell death.^25^ Our study demonstrated that MBP significantly increased the levels of ROS in TM3 cells, reduced the expression of antioxidant enzymes CAT, SOD1, and SOD2, and induced cellular oxidative stress. Furthermore, BPA and MBP significantly elevated the expression of UPR markers GRP94 and GRP78. GRP94 and GRP78 are hallmark molecular chaperones in the endoplasmic reticulum that play vital roles in protein folding, assembly, and transport under conditions of cellular stress. Following endoplasmic reticulum dysfunction, the expression of GRP94 and GRP78 increases significantly, triggering the recognition and transduction of UPR signals via transmembrane signaling proteins, which activate the ERS signaling pathway.^26^ Currently, three key signaling pathways involved in ERS activation have been primarily identified: PERK, IRE1, and ATF6.^27^ Interestingly, we found that MBP activated the PERK/EIF2α/ATF4/CHOP pathway in TM3 cells. During ERS, PERK is activated and inhibits protein translation by phosphorylating EIF2α, thereby reducing the workload on the ER.^28^ However, when ERS persists, CHOP activates, recruiting extrinsic apoptotic receptors via death ligands (Fas, TNF-α, and Trail), which, in turn, activate cytoplasmic caspase-8 and downstream caspase-3, ultimately inducing apoptosis.^29^ In the present study, after ERS in Leydig cells, we also observed a significant increase in the number and proportion of TUNEL-positive cells in the BPA and MBP groups, as well as elevated levels of cleaved caspase-3 and increased apoptosis rates, indicating that BPA and MBP may promote apoptosis through oxidative stress and ERS.

MT is a well-known indoleamine primarily synthesized in the pineal gland. Beyond its role in regulating sleep, MT is recognized for its potent antioxidant properties, which eliminate accumulated free radicals, enhance antioxidant enzyme activity, and thereby mitigate cellular oxidative stress, reduce DNA mutations, and prevent apoptosis.^30^ MT promotes mitophagy through the SIRT1/FoxO1 signaling pathway, alleviating oxidative stress-induced apoptosis in bovine ovarian granulosa cells.^11^ It also inhibits doxorubicin-induced oxidative stress and apoptosis in cardiomyocytes by activating the Sirt1/Nrf2 pathway.^14^ Additionally, MT upregulates SIRT3 to inhibit oxidative stress, enhancing the activity of the antioxidant enzyme SOD2 and suppressing apoptosis in intestinal epithelial cells.^15^ Our research demonstrated that MT significantly reduced the levels of ROS elevated by MBP in TM3 cells and increased the expression of antioxidant enzymes SOD1, SOD2, and CAT, thereby significantly decreasing MBP-induced apoptosis in TM3 cells. The attenuation of oxidative stress may be one of the mechanisms through which MT mitigates apoptosis in Leydig cells of the testes.

MT not only alleviates cellular oxidative stress through its antioxidant properties but also serves as a protective agent against ERS.^13^, ^31^ ERS activates the UPR, aiming to restore normal endoplasmic reticulum function; however, if the stress remains unresolved, it may trigger apoptosis.^32^ In neurodegenerative diseases, MT has been shown to target CHOP, PERK, and GRP78/BiP molecules, thus alleviating ERS in nerve cells and inhibiting neuronal apoptosis.^12^ Additionally, MT improves ERS in hepatocytes via the MT2/cAMP/PKA/IRE1 pathway, thereby inhibiting hepatocyte necrosis induced by non-alcoholic fatty liver disease.^13^ Furthermore, MT regulates ATF4 to activate the mTOR signaling pathway in follicular granulosa cells, suppressing the expression of ERS-related genes GRP78, CHOP, PERK, EIF-2α, and BAX, thereby inhibiting apoptosis in these cells.^33^ Mechanistically, unresolved or persistent ERS promotes the phosphorylation of PERK, which in turn phosphorylates its downstream substrate EIF2α. Although this phosphorylation initially serves as an adaptive mechanism to reduce global protein synthesis, sustained EIF2α phosphorylation paradoxically promotes the selective translation of ATF4 mRNA.^34^ The accumulated ATF4 then translocates into the nucleus and transcriptionally upregulates the expression of CHOP, a key pro-apoptotic transcription factor.^35^ CHOP subsequently promotes the transcription of pro-apoptotic factors such as BAX and BAK, leading to mitochondrial outer membrane permeabilization and the release of cytochrome c. Cytochrome c interacts with apoptotic protease activating factor-1 (Apaf-1) to form the apoptosome, which in turn activates the caspase cascade.^35^ Ultimately, this cascade converges on the cleavage and activation of caspase-3, the principal executioner protease responsible for dismantling cellular structures and driving cells irreversibly toward apoptosis.^36^ Consistent with this mechanism, in our study, MT reduced the expression of GRP94 and GRP78, inhibited the PERK/EIF2α/CHOP signaling pathway, and decreased cleaved caspase-3 as well as the cleaved caspase-3/total caspase-3 ratio in both BPA-treated mice and MBP-treated TM3 cells; in addition, ATF4, a downstream effector of this pathway, was also downregulated by MT in MBP-treated TM3 cells. These results indicate that the protective effects of MT against ERS and apoptosis are reproducible from the *in vivo* BPA model to the *in vitro* MBP model. The suppression of ERS in Leydig cells by MT may represent one of the mechanisms through which it inhibits apoptosis.

SIRT1 is an NAD^+^-dependent deacetylase that plays a significant role in cellular responses to inflammation, metabolism, and oxidative stress.^11^ Recent studies have identified SIRT1 as a crucial regulatory molecule in cellular ERS in various cell types, where it modulates ERS through the PERK pathway.^37^, ^38^, ^39^, ^40^ Research has demonstrated that SIRT1 promotes the deacetylation of PERK, thereby reducing the activation of the UPR-induced PERK-EIF2α-CHOP pathway.^41^ Additionally, SIRT1 has been shown to directly induce deacetylation of lysine K143 on EIF2α, thereby protecting cardiomyocytes from ERS damage.^39^ MT can relieve isoflurane-induced ERS and apoptosis in hippocampal neurons via the SIRT1/Mfn2/PERK pathway.^38^ Similarly, in the present study, we found that MT activated SIRT1 in TM3 cells through multiple mechanisms: upregulating total protein expression, promoting nuclear retention, and enhancing deacetylase activity. Furthermore, MT inhibited the ERS-PERK/EIF2α/ATF4/CHOP pathway and significantly reduced cell apoptosis caused by BPA or MBP, and these effects were attenuated by the SIRT1 inhibitor EX527. These findings indicate that MT exerts protective effects against ERS induced by BPA or MBP at least partially through activation of the SIRT1 pathway.

Although the present study demonstrates that MT increases SIRT1 protein expression and enhances its nuclear localization and deacetylase activity, the upstream molecular mechanisms by which MT upregulates SIRT1 remain to be elucidated. SIRT1 can be modulated at multiple levels, including transcriptional regulation, post-translational modifications, and protein stability.^42^, ^43^, ^44^ Future studies investigating these possibilities—including qRT-PCR for SIRT1 mRNA, cycloheximide chase assays for protein half-life, and ubiquitination assays—will be essential to fully understand how MT orchestrates SIRT1-mediated protection against ERS. An unexpected observation was that EX527 treatment reduced SIRT1 protein levels in TM3 cells. While EX527 is characterized as a selective inhibitor of SIRT1 deacetylase activity, accumulating evidence suggests that SIRT1 activity and protein stability may be interconnected through feedback regulatory mechanisms. For instance, Liu et al. demonstrated that the deubiquitinase USP28 stabilizes SIRT1 via deubiquitination, an effect reversed by EX527.^45^ Consistent with our findings, EX527-induced downregulation of SIRT1 protein has been reported across multiple experimental systems.^46^, ^47^, ^48^, ^49^, ^50^ These observations suggest that under conditions of cellular stress, SIRT1 activity and protein abundance may be coupled through regulatory loops. Nevertheless, the precise mechanism by which EX527 reduces SIRT1 protein levels in Leydig cells under MBP-induced stress remains to be elucidated.

The primary function of testicular Leydig cells is to synthesize and secrete androgens to promote spermatogenesis. Testosterone synthesis begins when cholesterol is transported into the mitochondria by the StAR (steroidogenic acute regulatory) protein. Within the mitochondria, CYP11A1 converts cholesterol to pregnenolone, which is then transferred to the smooth endoplasmic reticulum, where it is further converted into testosterone by essential steroidogenic enzymes, including 3β-HSD, CYP17A1, and 17β-HSD.^51^ These enzymes, located in both mitochondria and the endoplasmic reticulum, are crucial for testosterone synthesis and the maintenance of Leydig cell function. When testicular Leydig cells are subjected to oxidative stress or ERS damage, the expression of these steroidogenic enzymes is affected, inhibiting testosterone synthesis and secretion, and subsequently impacting spermatogenesis and sperm maturation.^52^, ^53^ Research has investigated substances such as Apigenin,^14^ Quercetin,^54^ and Geniposide^55^ for their potential to alleviate ERS damage in testicular Leydig cells caused by obesity, diabetes, and cyclophosphamide, thereby enhancing testosterone synthesis and secretion and maintaining normal function of the seminiferous tubules. However, there is limited research on the potential mitigating effects of MT on BPA- or MBP-intervened testosterone synthesis in Leydig cells. Our study found that in mice treated with BPA or MBP, Leydig cells experiencing oxidative stress and ERS damage exhibited significantly reduced testosterone synthesis and secretion, along with decreased expression of the steroidogenic enzymes CYP17A1 and 17β-HSD. Notably, treatment with MT significantly increased the expression of CYP17A1 and 17β-HSD and enhanced testosterone secretion in TM3 cells. Consistently, studies have demonstrated that MT can regulate the expression of steroidogenic enzymes such as StAR, p450scc/CYP11A1, and 3β-HSD,^56^, ^57^ thereby promoting testosterone synthesis. Additionally, after MT treatment in BPA-intervened mice, the seminiferous epithelium of the testicular tubules exhibited significant thickening, an orderly arrangement of germ cells, and a notable increase in the density and viability of epididymal sperm, all closely associated with the recovery of Leydig cell function. This further verifies the protective effects of MT on Leydig cells.

It is worth noting that MT can exert its biological effects through both receptor-dependent (via membrane receptors MT1 and MT2) and receptor-independent mechanisms (such as direct free radical scavenging and modulation of intracellular enzymes). Our previous study demonstrated that BPA exposure downregulated MT receptor 1A expression in Leydig cells, and this effect was reversed by MT treatment,^5^ suggesting a potential receptor-mediated component in the protective action of MT. However, the present study focused on intracellular signaling pathways downstream of receptor activation or independent of receptors and did not experimentally dissect the relative contributions of receptor-dependent versus receptor-independent mechanisms. Future studies using MT receptor antagonists or receptor-knockout models will be necessary to determine whether the observed PERK pathway modulation requires MT receptor activation.

We recognize several limitations in the present study. First, the BPA dose administered to mice (50 mg/kg/day) is substantially higher than typical human environmental exposure levels. This dose was chosen to establish a reproducible testicular injury model within a feasible 30-day treatment period. However, this design does not directly model chronic, low-dose human exposure scenarios. Long-term studies are necessary to determine whether MT confers sustained protection against cumulative reproductive damage. Second, while the TM3 Leydig cell line is a widely used *in vitro* model, it may not fully recapitulate the physiology of primary Leydig cells. Future validation using primary human Leydig cells or testis organoid models is necessary. Third, the current study lacks direct genetic evidence, such as SIRT1 knockdown or overexpression experiments, to definitively establish that MT acts through SIRT1. Relying solely on the pharmacological inhibitor EX527 is a limitation, as inhibitors may have off-target effects. In addition, apoptosis in this study was mainly assessed by TUNEL and caspase-3 activation (including the cleaved caspase-3/total caspase-3 ratio), complemented by Annexin V/PI flow cytometry. Other apoptosis-related markers, such as Bax, Bcl-2, PARP, caspase-8, and caspase-9, were not examined. Future studies involving SIRT1 genetic manipulation and a broader panel of apoptotic markers will be necessary to establish the causal role of SIRT1 and to further define the apoptotic pathways involved. Fourth, using single concentrations without full dose-response curves may not exclude potential off-target effects. Future studies incorporating systematic dose-response analyses will be important. Fifth, although MT restores testicular histology and sperm parameters, fertility restoration was not directly assessed. Direct fertility tests are required to confirm functional reproductive recovery. Ongoing studies in our laboratory are addressing these questions using chronic exposure models, primary cell cultures with SIRT1 genetic manipulation, and fertility trials.

## Conclusion

In conclusion, BPA exposure poses a significant risk to male reproductive health. This study elucidates the protective role of MT against damage to testicular Leydig cells induced by BPA and its metabolite MBP. The results demonstrate that MT alleviates oxidative stress in testicular Leydig cells caused by BPA and MBP, and further mitigates ERS in Leydig cells by regulating the PERK pathway, inhibiting apoptosis, and promoting testosterone synthesis, thereby improving the function of testicular seminiferous tubules and the maturation of epididymal sperm. The alleviation of MBP-induced ERS by MT may be associated with SIRT1 activation, as supported by our EX527 inhibitor experiments, ATF4 data, and SIRT1 nuclear localization and activity studies. These results support the potential of MT as a promising therapeutic agent for alleviating testicular injuries caused by environmental pollutants, warranting further exploration of its clinical significance in male reproductive health.

## CRediT authorship contribution statement

**Qi Qi:** Writing – original draft, Validation, Resources, Methodology, Investigation, Formal analysis, Data curation. **Jijing Xu:** Writing – review & editing, Methodology, Investigation, Funding acquisition, Formal analysis. **Shuang Li:** Writing – review & editing, Software, Investigation, Formal analysis. **Xiaoyan Pan:** Writing – review & editing, Validation, Supervision, Project administration, Data curation, Conceptualization.

## Declarations of interest

The authors declare that they have no known competing financial interests or personal relationships that could have appeared to influence the work reported in this paper.

## Data Availability

I have shared the link to my data at the attach file step FigshareMelatonin Mitigates Bisphenol A- and MBP-Induced Damage in Leydig Cells by Alleviating Oxidative Stress and Endoplasmic Reticulum Stress via inhibition of PERK signaling pathway
