# Supplementary material for: Melatonin mitigates bisphenol A- and MBP-induced damage in Leydig cells by alleviating oxidative stress and endoplasmic reticulum stress via inhibition of PERK signaling pathway
Source: Cell Stress Chaperones. 2026 Oct 15;31(6):100222. doi: 10.1016/j.cstres.2026.100222 (PMC13721099; doi:10.1016/j.cstres.2026.100222)
Supplement: Supplementary file 1 [file mmc1.docx]

**Supplementary Material. Full, uncropped Western blot images for all key proteins (n = 3). The blots correspond to the cropped images presented in Figures 2, 4, 8, 9, 10, and 11.**

**Original blots for Figure 2E**

**Original blots for Figure 4E**

**Original blots for Figure 8A**

**Original blots for Figure 8E**

**Original blots for Figure 8G**

**Original blots for Figure 9A**

**Original blots for Figure 9B**

**Original blots for Figure 9C**

**Original blots for Figure 9D**

**Original blots for Figure 9E**

**Original blots for Figure 9F**

**Original blots for Figure 10A**

**Original blots for Figure 10C**

**Original blots for Figure 11A**

**Original blots for Figure 11B**

**Original blots for Figure 11C**

原始图标注的字号太小了，看不清楚，建议调大

**Original blots for Figure 4E**
